# The association of sociodemographic differences with clinical management following a diagnosis of short cervix

**DOI:** 10.1002/pmf2.70278

**Published:** 2026-03-26

**Authors:** Joseph R. Biggio, Paula L. McGee, George R. Saade, Rebecca G. Clifton, Monica Longo, Sabine Z. Bousleiman, Kelly Clark, William A. Grobman, Heather A. Frey, Suneet P. Chauhan, Lorraine Dugoff, Tracy A. Manuck, Edward K. Chien, Dwight J. Rouse, Hyagriv N. Simhan, M. Sean Esplin, George A. Macones, Matthew K. Hoffman, for the Eunice Kennedy Shriver National Institute of Child Health and Human Development Maternal‐Fetal Medicine Units (MFMU) Network

**Affiliations:** ^1^ Division of Maternal Fetal Medicine, Department of Obstetrics and Gynecology University of Alabama at Birmingham Heersink School of Medicine Birmingham Alabama USA; ^2^ The George Washington University Biostatistics Center Washington District of Columbia USA; ^3^ Division of Maternal Fetal Medicine, Department of Obstetrics and Gynecology University of Texas Medical Branch at Galveston Galveston Texas USA; ^4^ *Eunice Kennedy Shriver* National Institute of Child Health and Human Development Bethesda Maryland USA; ^5^ Division of Maternal Fetal Medicine, Department of Obstetrics and Gynecology Columbia University New York New York USA; ^6^ Division of Maternal Fetal Medicine, Department of Obstetrics and Gynecology University of North Carolina at Chapel Hill Chapel Hill North Carolina USA; ^7^ Division of Maternal Fetal Medicine, Department of Obstetrics and Gynecology Northwestern University Chicago Illinois USA; ^8^ Division of Maternal Fetal Medicine, Department of Obstetrics and Gynecology The Ohio State University Columbus Ohio USA; ^9^ Division of Maternal Fetal Medicine, Department of Obstetrics, Gynecology and Reproductive Sciences University of Texas Health Science at Houston McGovern Medical School Houston Texas USA; ^10^ Division of Maternal Fetal Medicine, Department of Obstetrics and Gynecology Hospital of the University of Pennsylvania Philadelphia Pennsylvania USA; ^11^ Division of Maternal Fetal Medicine, Department of Reproductive Biology Case Western Reserve University Cleveland Ohio USA; ^12^ Division of Maternal Fetal Medicine, Department of Obstetrics and Gynecology Brown University Providence Rhode Island USA; ^13^ Division of Maternal Fetal Medicine, Department of Obstetrics, Gynecology, and Reproductive Sciences The University of Pittsburgh Medical Center Magee‐Womens Hospital Pittsburgh Pennsylvania USA; ^14^ Division of Maternal Fetal Medicine, Department of Obstetrics and Gynecology University of Utah Salt Lake City Utah USA; ^15^ Division of Maternal Fetal Medicine, Department of Women's Health University of Texas at Austin Dell Medical School Austin Texas USA

**Keywords:** pelvic rest, preterm birth, short cervix, sociodemographic differences, systemic bias

## Abstract

**Introduction:**

Following diagnosis of a short cervix from 16 through 23 weeks there is considerable variation in clinical practice. We sought to evaluate the association of patient sociodemographic factors with variation in clinical management following diagnosis of a short cervix.

**Methods:**

This was a non‐prespecified secondary analysis of the randomized Trial of Cervical Pessary for Short Cervix in Singleton Pregnancies (TOPS), in which individuals with singleton pregnancies with a cervical length (CL) ≤20 mm were randomized to either a pessary or usual care. We assessed recommendations for vaginal progesterone, additional CL surveillance, pelvic rest, bed rest, and cerclage. For individuals enrolled in the trial, additional CL assessment and cerclage were discouraged in the absence of significant cervical dilation; activity modification and vaginal progesterone were at provider's discretion. Sociodemographic factors assessed were self‐reported race and ethnicity, marital status, employment status, years of education, and type of insurance. Multivariable regression models examined the association between each sociodemographic factor and management, adjusting for other sociodemographic factors, CL at enrollment, clinical center, and treatment arm; odds ratios (ORs) and 95% confidence intervals (CIs) were determined.

**Results:**

Of the 446 patients (82% of 544 enrolled in TOPS) with at least one post enrollment visit, 95.5% received vaginal progesterone, 28.0% had at least one additional CL assessment, 55.8% were placed on pelvic rest, 19.5% on activity restriction, and 7.4% underwent cerclage. The high rate of vaginal progesterone usage precluded evaluation of association with sociodemographic factors. Non‐Hispanic Black and Hispanic patients had lower odds of repeat CL assessment compared with Non‐Hispanic White patients (adjusted OR [aOR], 0.47; 95% CI, 0.24, 0.91; aOR, 0.45; 95% CI, 0.21, 0.99, respectively). No other sociodemographic factors were associated with repeat CL. Patients with private insurance, but no other factor, had higher odds of pelvic rest being recommended (aOR, 2.08; 95% CI, 1.25, 3.45). None of the examined sociodemographic factors were associated with recommendations for bed rest or cerclage.

**Conclusion:**

Following diagnosis of a short cervix, vaginal progesterone is prescribed for nearly all patients. Non‐Hispanic Black and Hispanic patients were less likely to have a repeat CL assessment following diagnosis of a short cervix and those with private insurance were more likely to have pelvic rest recommended. Variation in the frequency of these interventions may reflect how sociodemographic and socially constructed factors can affect clinical decision making and implementation of interventions in the absence of definitive guidance.

**Trial registration:**

ClinicalTrials.gov Identifier: NCT02901626.

## 1 INTRODUCTION

Cervical length (CL) measurement in the midtrimester has become increasingly common as part of routine obstetric care in patients with singleton gestations and no prior history of spontaneous preterm birth in an effort to identify those at risk for preterm birth. Following the identification of a short CL on transvaginal ultrasound, the most effective approach to treatment remains a matter of debate. The American College of Obstetricians and Gynecologists (ACOG) and the Society for Maternal‐Fetal Medicine (SMFM) recommend initiation of vaginal progesterone [1, 2], although whether a CL of 20 or 25 mm should be the threshold at which this is recommended is not clear [3]. After the initiation of progesterone, there is a lack of evidence and consensus on the utility of further CL assessment and the role of any additional interventions, including cerclage placement or activity restriction [2, 4, 5, 6, 7].

In the recently completed Trial of Cervical Pessary for Short Cervix in Singleton Pregnancies (TOPS), pessary did not reduce the rate of preterm delivery in patients with a singleton gestation and a CL of 20 mm or less [8]. Following enrollment in the study, there was considerable variation in management recommendations after identification of a short cervix despite the uncertainty of their efficacy. A variety of factors can influence decisions regarding interventions in the absence of evidence. Patient desire to “do something” and the physician belief that the patient “wants something done” can drive such decisions [9]. Such perceptions can be based on unconscious and systemic bias that result in differences in recommendations based on beliefs regarding which interventions a patient might adhere to, desire, or need [10]. In obstetrics, implicit and systemic bias have been shown to contribute to disparities in several outcomes and interventions including pain control, timely treatment of hypertension, and cesarean rates [11]. However, data examining associations between sociodemographic factors and implementation of interventions in the setting of cervical shortening are lacking.

Given the lack of evidence to support recommendations for follow‐up and interventions seen in the TOPS trial, we sought to evaluate to what extent sociodemographic differences were associated with variation in recommendations for antenatal management following diagnosis of a short cervix and enrollment in the trial.

## 2 METHODS

This was a secondary analysis of the TOPS trial, the details of which have been previously published [8]. Briefly, the TOPS trial was a multicenter study conducted by the *Eunice Kennedy Shriver* NICHD Maternal‐Fetal Medicine Units Network to examine the efficacy of the Arabin cervical pessary for the prevention of preterm birth in patients with a singleton gestation, no history of preterm birth, and a transvaginal CL of 20 mm or less. Participants were screened at 16 weeks 0 days to 23 weeks 6 days, and those qualifying were randomized to either pessary placement or expectant management. The trial protocol did not mandate any other interventions or treatment. Specifically, progesterone initiation and activity restriction were at the provider's discretion. Follow‐up CL measurement was discouraged as it is not easily performed with a pessary in place, attempts at subsequent measurement in only the expectant management arm could lead to bias in trial outcomes, and there is no measurement at which different management would be clearly indicated. Cerclage was discouraged in the absence of cervical dilation regardless of CL. All patients who enrolled in the TOPS trial and who had at least one follow‐up study visit were included in this analysis.

For this study, we examined the frequency of interventions after enrollment at each scheduled monthly study visit, including treatment with vaginal progesterone (any formulation), performance of repeat CL assessment, placement of cerclage, and any recommendation for bed rest and/or pelvic rest. At each study visit, participants were asked whether there had been any change in physician management recommendations since the prior visit and data on the performance of CL assessment or cerclage placement were collected. The association of each intervention and the sociodemographic variables collected during the trial was evaluated. Sociodemographic variables included were self‐reported race/ethnicity (Hispanic/Non‐Hispanic, Black, White, with all others grouped together due to small numbers); marital status (married or living with partner vs. not); employment status (any vs. none); education level (≥12 years vs. <12 years of education); and payor status (private insurance or other).

Logistic regression models with a random intercept for clinical center and an independent covariance structure were used to examine the association between each sociodemographic factor and recommendation. Models were estimated both unadjusted and adjusted for other sociodemographic factors, CL at enrollment, and randomized treatment arm; odds ratios (ORs) and 95% confidence interval (CI) were determined. All statistical models were assessed for collinearity among predictor variables. There were no adjustments for multiple comparisons as this was a secondary analysis that was aimed to be hypothesis‐generating as opposed to hypothesis‐proving. All analyses were performed using SASv9.4.

## 3 RESULTS

There were 544 participants enrolled in the TOPS trial, of whom 446 (82%) had at least one monthly post‐enrollment study visit (Table 1). The median gestational age at randomization in TOPS was 21.7 weeks with a first monthly visit anticipated at 25–26 weeks; 95 participants (21%) delivered prior to 26 weeks. Delivery was the primary reason for lack of a follow‐up visit. Although not mandated by the protocol, 426 (96%) received some form of vaginal progesterone. Utilization of other interventions was less frequent, but not uncommon. A total of 125 (28%) had at least one additional CL measurement, 249 (56%) had documented pelvic rest recommended, 87 (20%) had documented bed rest recommended, and 33 (7%) had a cerclage placed (Table 2).

**TABLE 1:** Baseline characteristics.

	(*N* = 446) *n* (%)
Maternal age, mean (SD)	29.6 (5.9)
Race ethnicity	
Non‐Hispanic Black	208 (46.6)
Non‐Hispanic White	98 (22.0)
Hispanic	99 (22.2)
Asian	23 (5.2)
American Indian	1 (0.2)
Other/not reported/unknown	17 (3.8)
Married/living with partner	275 (61.7)
Employed full or part time	300 (67.3)
More than 12 years of school	277 (62.1)
Insurance	
Self‐pay	12 (2.7)
Private	237 (53.1)
Government	197 (44.2)

**TABLE 2:** Sociodemographic characteristics with recommendations for interventions following diagnosis of a short cervix.

	Progesterone use (*n* = 426)	Repeat CL ultrasound (*n* = 125)	Pelvic rest (*n* = 249)	Bed rest (*n* = 87)	Cerclage (*n* = 33)
	*n* (%)	*n* (%)	*n* (%)	*n* (%)	*n* (%)
Race and ethnicity					
Non‐Hispanic White (*n* = 98)	96 (98.0)	36 (36.7)	60 (61.2)	19 (19.4)	10 (10.2)
Non‐Hispanic Black (*n* = 208)	193 (92.8)	51 (24.5)	106 (51.0)	38 (18.3)	11 (5.3)
Hispanic (*n* = 99)	96 (97.0)	25 (25.3)	57 (57.6)	22 (22.2)	7 (7.1)
Asian/American Indian/Other/not reported/unknown (*n* = 41)	41 (100)	13 (31.7)	26 (63.4)	8 (19.5)	5 (12.2)
Married/living with partner					
Yes (*n* = 275)	264 (96.0)	72 (26.2)	163 (59.3)	59 (21.5)	23 (8.4)
No (*n* = 171)	162 (94.7)	53 (31.0)	86 (50.3)	28 (16.4)	10 (5.9)
Employed					
Yes (*n* = 300)	289 (96.3)	81 (27.0)	166 (55.3)	62 (20.7)	21 (7.0)
No (*n* = 146)	137 (93.8)	44 (30.1)	83 (56.9)	25 (17.1)	12 (8.2)
More than 12 years of school					
Yes (*n* = 277)	268 (96.8)	71 (25.6)	162 (58.5)	61 (22.0)	24 (8.7)
No (*n* = 169)	158 (93.5)	54 (32.0)	87 (51.5)	26 (15.4)	9 (5.3)
Private insurance					
Yes (*n* = 237)	229 (96.6)	67 (28.3)	150 (63.3)	49 (20.7)	22 (9.3)
No (*n* = 209)	197 (94.3)	58 (27.8)	99 (47.4)	38 (18.2)	11 (5.3)

**FIGURE 1:**
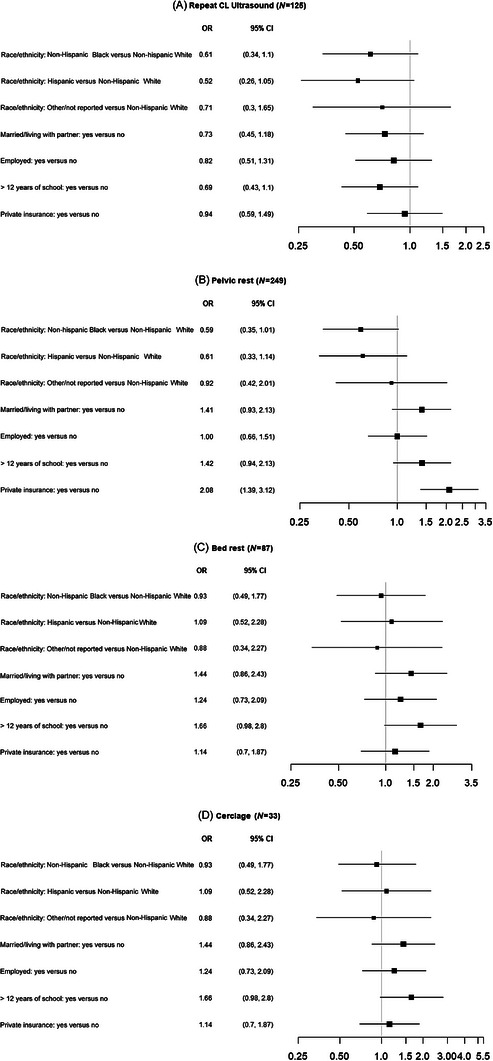
Unadjusted association of sociodemographic characteristics with recommendations for interventions following diagnosis of a short cervix. Logistic models with a random intercept for clinical center. An odds ratio (OR) of 1.0 indicates equal odds of receiving the management/recommendation between the two groups. (A) Repeat cervical length assessment; (B) pelvic rest; (C) bed rest; (D) cerclage. CI, confidence interval.

**FIGURE 2:**
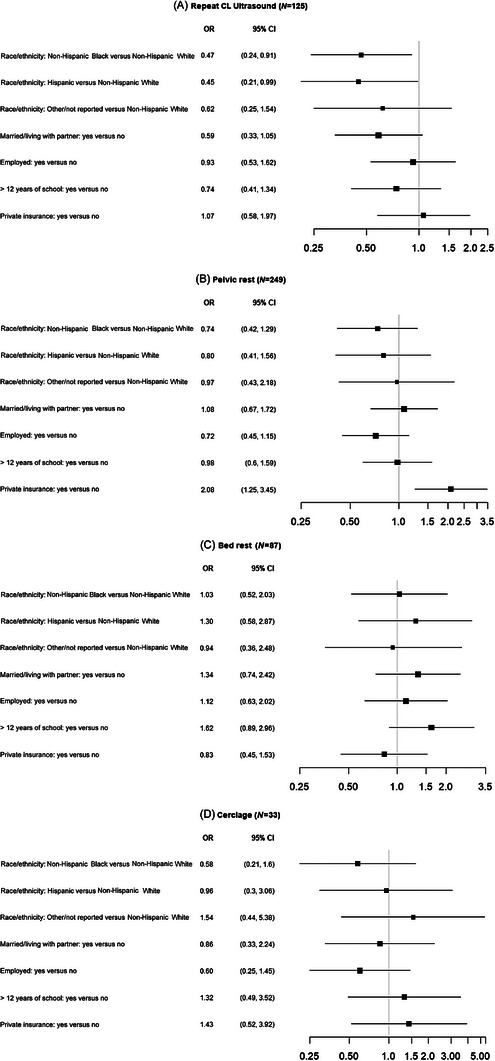
Adjusted association of sociodemographic characteristics with recommendations for interventions following diagnosis of a short cervix. Logistic models adjusted for race and ethnicity, marital status, employment, education, medical payment, cervical length at enrollment, and treatment group with a random intercept for clinical center. An odds ratio (OR) of 1.0 indicates equal odds of receiving the management/recommendation between the two groups. (A) Repeat cervical length assessment; (B) pelvic rest; (C) bed rest; (D) cerclage. CI, confidence interval.

The near universal use of vaginal progesterone in both the pessary and expectant management arms of the study precluded meaningful evaluation of the association of sociodemographic factors with use of progesterone (Table 2). Repeat CL measurement was performed less commonly in Non‐Hispanic Black (25%) and Hispanic patients (25%) compared to Non‐Hispanic White patients (37%) (adjusted [aOR], 0.47; 95% CI, 0.24, 0.91; aOR, 0.45; 95% CI, 0.21, 0.99, respectively) after adjustment for other covariates. No other sociodemographic factors were associated with repeat CL assessment (Figures 1 and 2, Table 2). Patients with private insurance were more likely to receive a recommendation for pelvic rest compared to those with governmental insurance or who were self‐pay (63% vs. 47%; aOR, 2.08; 95% CI, 1.25, 3.45) after adjustment for other covariates, with a similar unadjusted association (OR 2.08; 95% CI, 1.39, 3.12). None of the other examined sociodemographic variables were associated with recommendations for pelvic rest. Similarly, there was no association between recommendations for bed rest or placement of cerclage with any of the sociodemographic factors examined both in the unadjusted and adjusted models.

## 4 DISCUSSION

In the setting of a multicenter clinical trial of pessary use in the setting of a short cervix, conducted at medical centers across the country, there was a near universal rate of vaginal progesterone prescription. Multiple studies and meta‐analyses have demonstrated a 35%–45% reduction in preterm birth with vaginal progesterone following the identification of cervical shortening [3, 12, 13], and it is the only intervention for this indication recommended by SMFM and ACOG [1, 2]. Given these recommendations, it was reassuring to see that the rate of utilization of vaginal progesterone was similar regardless of sociodemographic factors.

Although repeat CL assessment can identify patients with progressive cervical shortening following initiation of vaginal progesterone, there is a lack of consensus on the value of such surveillance or the effectiveness of additional treatments, including cerclage [1, 2, 4, 5, 6, 7]. While there is some evidence to suggest a potential role for cerclage in patients who become symptomatic and/or develop cervical dilation [5], the quality of evidence is low and insufficient and neither ACOG nor SMFM recommend further CL surveillance or cerclage in the absence of symptoms [1, 2]. Despite the lack of recommendations for repeat CL and active discouragement through participation in the TOPS trial, we found that repeat CL measurement was more than 50% less likely to occur in Non‐Hispanic Black and Hispanic patients when compared to Non‐Hispanic White patients after controlling for other variables. The difference in physician management may reflect structural factors that impact access to interventions, for example, transportation and time off work, or may reflect unconscious or systemic bias, including beliefs regarding risks to the patient, patient adherence to recommendations and understanding of risk, and beliefs regarding the patient's desire for further follow‐up and intervention [9, 10].

No form of activity restriction, including bed rest and pelvic rest, has been shown to prolong pregnancy or prevent preterm birth in patients with a short cervix. Accordingly, SMFM has recommended against such restrictions [14]. Nevertheless, just as seen for CL reassessment in this clinical study, pelvic rest was more than twice as likely to be recommended for patients with private insurance than for those with other payor statuses. The increased frequency of this recommendation in patients with private insurance may reflect a variety of nonclinical factors that have been associated with clinical decision‐making and affect evidence‐based practice including beliefs regarding adherence to or preference for recommendations as well as socioeconomic status [9, 10].

Implicit bias has been defined as attitudes that can influence actions, decisions, and communication in an unconscious fashion. Studies have demonstrated that members of the health care team demonstrate implicit bias in such a way that it impacts care and clinical communication due to certain assumptions regarding a patient attitude, desire, or adherence based on the group to which they belong [11]. In this current study, given the differences in the performance of CL screening and recommendations for pelvic rest, the possible impact of implicit bias, structural factors, and other nonclinical factors that affect care need to be considered [9, 10, 11].

The geographic diversity, the large number of medical centers across the United States that participated in the TOPS trial, and the diversity of the participant population are strengths of this study that enhance the generalizability of our findings. Because patients included in our analysis were enrolled in a clinical study, whether similar findings would occur outside the context of a clinical trial is unclear. There are also limitations to this study. We were limited in the sociodemographic variables we could examine to those collected in the primary study and thus cannot account for other potential sociodemographic factors, such as area deprivation index, food stability, and adverse childhood experiences, that could also be associated with differences in care due to systemic, structural, and other factors [15, 16, 17, 18]. We were unable to adjust for race and ethnicity of the physicians making clinical decisions, thereby limiting the ability to examine treatment recommendation based on race/ethnicity concordance. In addition to sociodemographic variables, a variety of other factors could influence the interventions offered or performed including patient preferences, institutional practice patterns, and information bias due to documentation style. We were unable to assess these factors. Also, this trial was performed primarily in academic medical centers, and as such, the findings may differ in other settings. Finally, there is the possibility that at least for some of the recommendations there may have been other indications, such as symptoms that were not captured in the study data but could explain a portion of the observed variation.

## 5 CONCLUSIONS

Among patients diagnosed with a short cervix, prescription of vaginal progesterone was nearly universal. There was considerable variation in other nonevidence‐based management strategies, including follow‐up CL assessment and recommendation for pelvic rest. Variation in the frequency of these interventions may reflect how sociodemographic, structural, socially constructed, and nonclinical factors can affect discretionary clinical decision‐making and implementation of interventions, especially in the absence of definitive guidance. Given the ongoing importance of achieving equity and access to the highest levels of evidence‐based practice for all patients, future work should focus on qualitative exploration of nonclinical drivers of care variation and ways to limit their impact at the patient, physician, and system level.

## AUTHOR CONTRIBUTIONS

Joseph R. Biggio designed this secondary analysis of the primary study. Matthew K. Hoffman, George R. Saade, Rebecca G. Clifton, Sabine Z. Bousleiman, Kelly Clark, William A. Grobman, Heather A. Frey, Suneet P. Chauhan, Lorraine Dugoff, Tracy A. Manuck, Edward K. Chien, Dwight J. Rouse, and George A. Macones were responsible for data collection and trial oversight of the primary study. Paula L. McGee performed data management and analyzed the data. Joseph R. Biggio wrote the first draft of the paper. All authors reviewed and had input to the manuscript.

## CONFLICT OF INTEREST STATEMENT

The authors declare no conflicts of interest.

## Data Availability

Deidentified participant data from the study will be available at the NICHD data repository (N‐DASH): https://dash.nichd.nih.gov/. Data sharing will be accessed and governed according to the procedures and policies of N‐Dash. A data dictionary will be provided. This data will be provided within 12 months of publication.
